# Serum levels of brain-derived neurotrophic factor (BDNF), proBDNF and plasma 3-methoxy-4-hydroxyphenylglycol levels in chronic schizophrenia

**DOI:** 10.1186/s12991-015-0084-9

**Published:** 2016-01-14

**Authors:** Reiji Yoshimura, Hikaru Hori, Asuka Katsuki, Kiyokazu Atake, Jun Nakamura

**Affiliations:** Department of Psychiatry, University of Occupational and Environmental Health, 1-1 Iseigaoka, Yahatanishi-ku, Kitakyushu, Fukuoka 8078555 Japan

**Keywords:** Brain-derived neurotrophic factor, 3-Methoxy-4-hydroxyphenylglycol, Chronic schizophrenia, Positive and negative symptoms score

## Abstract

**Background:**

We investigated the serum levels of brain-derived neurotrophic factor (BDNF), proBDNF and plasma 3-methoxy-4-hydroxyphenylglycol (MHPG) levels in patients with chronic schizophrenia.

**Methods:**

Sixty-eight patients who met the DSM-IV-TR criteria and had a chronic schizophrenia (CS) duration of ≥ 5 years were enrolled. Their serum brain-derived BDNF and proBDNF levels were measured by enzyme-linked immunosorbent assays, and their plasma MHPG levels were analyzed by high-performance liquid chromatography with electrochemical detection.

**Results:**

The plasma MHPG levels were significantly lower in the CS group (3.9  ±  1.8 ng/ml) compared to those in the group of 32 age- and sex-matched healthy controls (5.1  ±  2.4 ng/ml). The serum BDNF levels were significantly lower in the CS group (8.9  ±  4.8 ng/ml) compared to the control group (12.2.1  ±  6.8 ng/ml). No correlation was observed between plasma MHPG and BDNF in the CS group.

**Conclusion:**

These results suggest that hypo-activity of noradrenergic neurons and decreased BDNF secretion exist in chronic schizophrenic patients.

## Background

Although plasma 3-methoxy-4-hydroxyphenylglycol (MHPG) is used as a measure of peripheral catecholaminergic activity, an individual’s plasma MHPG level is also a good predictor of the central noradrenergic activity. We reported that plasma MHPG levels were lower in schizophrenia patients compared to those in healthy controls, and our findings demonstrated that risperidone significantly increased plasma MHPG levels, which was related to the improvement of negative symptoms of schizophrenia in a small sample [1]. We also reported that treatment with olanzapine for 4 weeks significantly increased plasma BDNF levels in acute schizophrenia patients [2]. Taking these findings into account, central noradrenergic dysfunctions may play a role in the pathogenesis of schizophrenia, especially negative symptoms and cognitive functions [3].

A recent meta-analysis showed that the plasma/serum brain-derived neurotrophic factor (BDNF) levels in schizophrenia patients were significantly lower than those in controls [4]. It is plausible that a decrease in synaptogenesis occurs in patients with schizophrenia. Taking these findings into account, we hypothesized that a reduced noradrenergic system and BDNF exist in schizophrenia patients.

Mature BDNF is initially synthesized as a precursor protein. ProBDNF is converted to mature BDNF by extracellular proteases. Mature BDNF is biologically active. In contrast, proBDNF, which is localized intracellularly, serves as an inactive precursor. In short, new evidence shows that proBDNF and mature BDNF elicit opposing effects via the p75NTR and TrkB receptors, respectively, and that both proBDNF and mature BDNF play important roles in several physiological functions of neurons, which might be related to the pathology of psychiatric disorders such as mood disorders and schizophrenia [5–9]. Briefly, mature BDNF is related to survival of neurons, promotion of growth, and differentiation of neurons [10, 11]. On the other hand, proBDNF activation includes promotion of myelination neuronal migration, neuronal process retraction, and neuronal apoptosis [12]. In 2012, Yoshida et al. [9] noted that it was initially thought that only secreted mature BDNF is biologically active, and that proBDNF serves as an inactive precursor. However, as mentioned above, proBDNF and BDNF appear to elicit opposing effects via the p75NTR and TrkB receptors, respectively, and both proBDNF and mature BDNF may play important roles in several physiological functions [12]. We recently reported that no correlation was however found between the Hamilton Rating Scale for Depression (HAMD17) scores and proBDNF. In addition, no relationship was found between the HAMD17 scores and the proBDNF/BDNF. Finally, the changes were observed serum levels of proBDNF and BDNF during the treatment durations with fluvoxamine, which indicates that no association exists between serum proBDNF/BDNF and fluvoxamine response in MDD patients at least within 4 weeks of the treatment [13]. From these findings, MHPG, mature- and proBDNF are important molecules for pathogenesis of schizophrenia.

To test our hypothesis, we investigated the relationship among the plasma levels of a noradrenaline metabolite (MHPG), serum BDNF levels, positive and negative syndrome scale (PANSS) scores, and global assessment of functioning (GAF) scores in patients with chronic schizophrenia. In addition, this is the first report investigating plasma proBDNF levels in patients with chronic schizophrenia.

## Methods

The subjects were 68 Japanese chronic schizophrenia patients (42 males and 26 females) treated at the University Hospital of the University of Occupational and Environmental Health, all of whom fulfilled the Diagnostic and Statistical Manual of Mental Disorders, fourth Edition, Text Revision (DSM-IV-TR) criteria for schizophrenia. The treatment durations were all ≥5 years. Their ages ranged from 33 to 70 (mean  ±  SD  =  48  ±  9) years. Their duration of illness ranged from 5 to 40 (mean  ±  SD  =  18  ±  9) years. Thirty-two age- and sex-matched healthy subjects (age: range 29–68 years, mean  ±  SD  =  47  ±  11 years; 20 males and 12 females) were also examined. All enrolled subjects did not have history of physical diseases; i.e., rheumatoid arthritis, hypertension, chronic kidney disease, cardiovascular disease, metabolic syndrome, cancer, atopic diseases, and the subjects did not take statins, aspirin, estradiol, dietary supplements such as vitamin E, vitamin A, vitamin B12, folic acid, zinc, omega-3 fatty acids, and ginko biloba.

The severity of the patients’ clinical symptoms was evaluated using the PANSS and GAF. The patients’ plasma concentrations of MHPG were analyzed by high-performance liquid chromatography with electrochemical detection (HPLC-ECD) as described [13]. Their serum levels of BDNF and proBDNF were assayed with a sandwich enzyme-linked immunosorbent assay (ELISA) as described [10].

The study protocol was approved by the Ethics Committee of the University of Occupational and Environmental Health. Written informed consent was obtained from all patients and controls.

### Statistical analysis

A non-paired *t* test was used to compare the plasma levels of MHPG and serum levels of BDNF or proBDNF between the chronic schizophrenia and control groups. The relationships between pairs of variables were examined using Person’s correlation coefficients. Significance of the results was set at *p* < 0.05.

## Results

### Medications of chronic schizophrenia patients

The medications of the chronic schizophrenia patients are listed in Table 1.

**Table 1:** Antipsychotic drugs prescribed for the patients

Antipsychotic drug	Range, dose (mean ± SD) mg/day	N of patients
Risperidone	2–8 (4.1 ± 2.3)	24
Olanzapine	5–20 (13.4 ± 6.1)	22
Aripiprazole	6–30 (17.4 ± 5.9)	24
Quetiapine	50–650 (372.6 ± 142.8)	8
Haloperidole	1–6 (2.4 ± 1.6)	2
Levomepromazine	50–200 (71.3 ± 49.2)	8
Zotepine	50–100 (69.4 ± 12.7)	2

### Plasma MHPG levels of the chronic schizophrenia patients and healthy controls

The plasma MHPG levels were significantly lower in the chronic schizophrenia (CS) group (4.4  ±  1.6 ng/ml) compared to the age- and sex-matched control group (6.8  ±  2.0 ng/ml) (*t*  =  −6.602, *p*  =  0.0000).

### Serum BDNF levels of the chronic schizophrenia patients and healthy controls

The serum BDNF levels were significantly lower in the CS group (10.2  ±  2.9 ng/ml) compared to the healthy controls (11.9  ±  4.3 ng/ml) (*t*  =  −2.447, *p*  =  0.016).

### Serum proBDNF levels of the chronic schizophrenia patients and healthy controls

No significant difference in serum proBDNF levels was revealed between the CS group (4.6  ±  2.1 ng/ml) and controls (4.7  ±  2.1 ng/ml) (*t*  =  −0.064, *p*  =  0.9491).

### Correlation between plasma MHPG levels and serum BDNF levels in the chronic schizophrenia patients

**Fig. 1:**
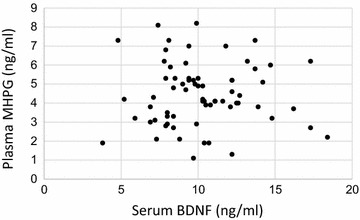
Correlation between plasma MHPG and serum BDNF

No correlation was observed between the plasma MHPG levels and serum BDNF levels in the CS patients (*r*  =  0.176, *p*  =  0.392) (Fig. 1).

### Correlation between plasma MHPG levels and serum proBDNF in the chronic schizophrenia patients

**Fig. 2:**
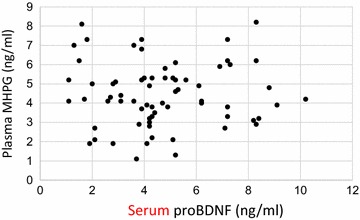
Correlation between plasma MHPG and proBDNF

No correlation exists between plasma MHPG levels and serum proBDNF levels in the chronic schizophrenia patients (*r*  =  0.099, *p*  =  0.641) (Fig. 2).

### No correlation between plasma MHPG levels and the PANSS scores

No correlation was observed between the plasma MHPG levels and any scores on the PANSS among the chronic schizophrenia patients (Table 2).

**Table 2:** Correlations between the serum BDNF and the PANSS scores

	BDNF	PANP	PANN	PANG	PANT
BDNF	1.0000	−0.051	0.0223	0.0848	0.024
PANP	−0.051	1.0000	−0.315	−0.253	0.4295
PANN	0.0223	−0.315	1.0000	0.1072	0.5413
PANG	0.0848	−0.253	0.1072	1.0000	0.4481
PANT	0.024	0.4295	0.5413	0.4481	1.0000

### No correlation between serum BDNF and the PANSS scores

No correlations were found between serum BDNF and any scores on the PANSS among the CS patients (Table 3).

**Table 3:** Correlations between the serum proBDNF level and the PANSS scores

	proBDNF	PANP	PANN	PANG	PANT
proBDNF	1.0000	0.0855	−0.062	−0.025	0.009
PANP	0.0855	1.0000	−0.315	−0.253	0.4295
PANN	−0.062	−0.315	1.0000	0.1072	0.5413
PANG	−0.025	−0.253	0.1072	1.0000	0.4481
PANT	0.009	0.4295	0.5413	0.4481	1.0000

### No correlation between serum proBDNF and the PANSS scores

No correlations were found between serum proBDNF and any scores on the PANSS among the chronic schizophrenia patients (Table 4).

**Table 4:** Correlations between the plasma MHPG levels and the PANSS scores

	MHPG	PANP	PANN	PANG	PANT
MHPG	1.0000	−0.199	0.0655	0.1339	−0.035
PANP	−0.199	1.0000	−0.315	−0.253	0.4295
PANN	0.0655	−0.315	1.0000	0.1072	0.5413
PANG	0.1339	−0.253	0.1072	1.0000	0.4481
PANT	−0.035	0.4295	0.5413	0.4481	1.0000

### No correlation between plasma MHPG and the GAF scores

No correlation was found between plasma MHPG (*r*  =  −0.104, *p*  =  0.292) and the GAF scores in the chronic schizophrenia group.

## Discussion

The main findings in the present study were that any correlations were not observed between serum BDNF, proBDNF, or plasma MHPG and the PANSS scores in the 68 chronic schizophrenia patients. No difference was found in the serum proBDNF between the two groups. Plasma levels of MHPG and the serum BDNF levels were significantly lower in the chronic schizophrenia patients compared to the healthy controls.

We reported in a preliminary study that plasma MHPG concentrations in patients with schizophrenia were lower than those in healthy subjects. The findings of the present study reconfirmed our preliminary results, especially the findings in the chronic schizophrenia patients. It is possible that noradrenergic activity decreases in chronic schizophrenia [14–16]. In addition, clozapine and risperidone treatment increased plasma noradrenaline levels. Taking these findings together, it appears that the hypo-activity of noradrenergic neurons may exist in chronic schizophrenia patients and atypical antipsychotic drugs may enhance noradrenergic functions. However, our present revealed no correlations between the plasma levels of MHPG or serum BDNF and any scores on the PANSS. One point at any situations of the patients and/or antipsychotic medications might explain the present results.

Chen et al. [17] reported that the application of noradrenaline in embryonic hippocampal neurons increased the serum levels of BDNF and phosphorylated tropomyosin receptor kinase (TrkB), and that these increases were prevented by extracellular signal-regulated kinase (ERK) and phosphatidylinositol 3-kinase inhibitors (PI-3K). They also showed that noradrenaline-induced increases in phospho-ERK2 and PI-3K were each suppressed by a PI-3K and mitogen-activated protein kinase inhibitor, respectively. In addition, the phosphorylation of cAMP-response element-binding protein (CREB) was also increased by noradrenaline and was reduced to baseline levels by MAPK and PI-3K inhibitors. In addition, both the MAPK and PI-3K inhibitors suppressed the phosphorylation of both TrkB and CREB. Taking all of these results into account, it appears that the noradrenaline-evoked BDNF expression leads a cyclic pathway, reminiscent of a positive feedback loop.

The results of another study elucidated an in vitro model of the intracellular signaling mechanisms activated by noradrenaline—via ligand G-protein-coupled receptor-to-BDNF-receptor tyrosine kinases transactivation — that is putatively thought to occur in vivo as a result of excitatory neural activity [18]. Astrocytes as an active part of the tripartite synapse can respond to the synoptically released neurotransmitters. BDNF is produced by astrocytes and neurons. Monoamines are able to potently and transiently increase BDNF cellular contents [19]. Taken together, these findings indicate a deficiency of noradrenaline brought to reduced BDNF levels in chronic schizophrenia; however, we observed no correlation between the plasma levels of MHPG and serum BDNF, which may be plausible that the noradrenaline function does not directly influence the BDNF level. In other words, it is possible that key molecules that regulate the balance between noradrenaline and BDNF might exist. We also observed that although treatment with an atypical antipsychotic drug increased plasma MHPG levels, it did not alter plasma BDNF levels [1, 2, 20].

The serum proBDNF level in chronic schizophrenia remains unknown. Yamamoto et al. [21] reported that matrix metalloproteinase-9, which plays a role in the conversion of proBDNF to mature BDNF, was significantly increased in patients with schizophrenia.

In addition, noradrenergic functions and BDNF were not related to the GAF scores of the present patients with chronic schizophrenia, indicating that both of the noradrenergic functions do not reflect the social activities of the chronic schizophrenia patients.

The present study has several limitations. This was a cross-sectional study, and all of the chronic schizophrenia patients were being treated with several medications; i.e., antipsychotics, mood stabilizers, and benzodiazepines. Thus, the medications might have influenced the results. Exercise, stress exposure, menstrual cycle, body mass index, and dietary composition also influenced the data. Finally, we did not check for the functional imaging of brain areas, where BDNF is concentrated.

In conclusion, any correlations were not found between serum BDNF, proBDNF, or plasma MHPG and the PANSS scores in the 68 chronic schizophrenia patients. We also reconfirmed that serum BDNF and plasma MHPG levels were significantly reduced in the 68 chronic schizophrenia patients compared to healthy controls.
